# Spatial redistribution of neurosecretory vesicles upon stimulation accelerates their directed transport to the plasma membrane

**DOI:** 10.1371/journal.pone.0264521

**Published:** 2022-03-16

**Authors:** Elaine B. Schenk, Frederic A. Meunier, Dietmar B. Oelz

**Affiliations:** 1 School of Mathematics & Physics, The University of Queensland, Brisbane, Australia; 2 Clem Jones Centre for Ageing Dementia Research, Queensland Brain Institute (QBI), The University of Queensland, Brisbane, Australia; UPR 3212 CNRS -Université de Strasbourg, FRANCE

## Abstract

Through the integration of results from an imaging analysis of intracellular trafficking of labelled neurosecretory vesicles in chromaffin cells, we develop a Markov state model to describe their transport and binding kinetics. Our simulation results indicate that a spatial redistribution of neurosecretory vesicles occurs upon secretagogue stimulation leading vesicles to the plasma membrane where they undergo fusion thereby releasing adrenaline and noradrenaline. Furthermore, we find that this redistribution alone can explain the observed up-regulation of vesicle transport upon stimulation and its directional bias towards the plasma membrane. Parameter fitting indicates that in the deeper compartment within the cell, vesicle transport is asymmetric and characterised by a bias towards the plasma membrane.

## Introduction

Neuroendocrine chromaffin cells in the adrenal medulla are a model system to study the exocytosis of secretory vesicles (SVs) which is preceded by their transport from the Golgi apparatus to their site of exocytosis on the plasma membrane [1]. Defects in this process have been tied to a range of neurodegenerative diseases [2] by mechanisms that are not yet fully elucidated [3]. The highly crowded and stochastic nature of the cytoplasm, however, represents a major difficulty in exploring intracellular transport systems and as a result many of the mechanisms involved in bringing vesicles towards the plasma membrane remain elusive [1].

Microtubules and actin filaments perform a vital role in the transport of secretory vesicles and can be considered the tracks of the intracellular transport network [4]. They are semi-flexible and some of them can be several μm in length.

In the central region of the cytoplasm both systems of cytoskeleton filaments, F-actin and microtubules, are involved in the cytoplasmic transport of secretory granules [5–7] through molecular motor proteins. In this cellular region microtubules are mostly aligned radially while close to the cortex their density is lower and they align tangentially with the cortex [6]. In the periphery of the cell, transport along the actin filament network occurs through processive molecular motors such as myosin-V [1, 8–10] and non-processive such as Myosin-II [11]. Recruitment of SVs to the cortical actin network occurs via the action of Myosin-VI short-insert isoform which has both a processive and a non-processive function [12].

**Fig 1:**
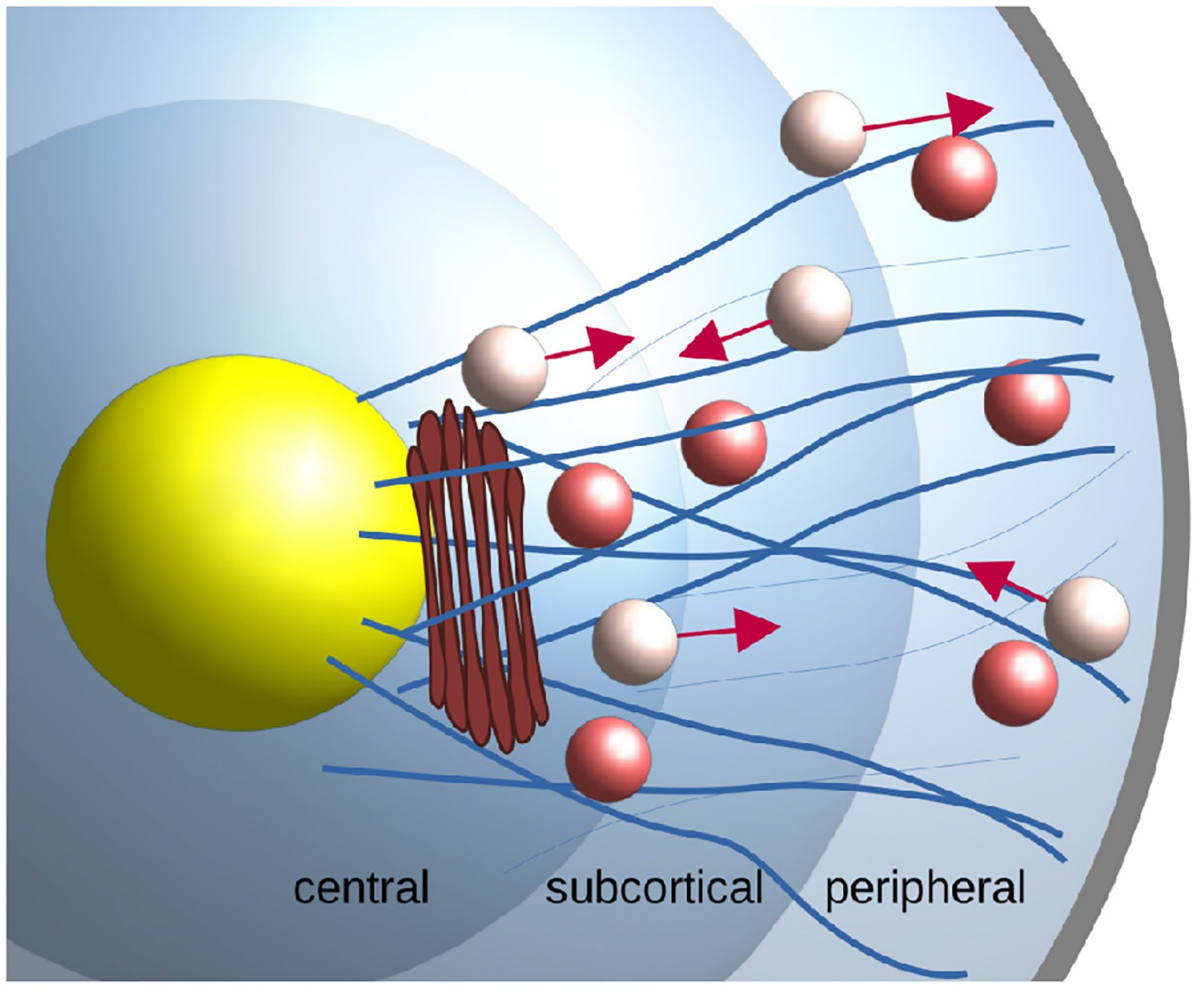
Sketch of secretory vesicle transport between the Golgi apparatus (brown) and the cortical actomyosin network in chromaffin cells (grey). Microtubules are shown as thick blue curves, actin filaments as thinner fibres. Secretory vesicle currently undergoing transport are represented by the lighter coloured spheres with arrows denoting the direction of transport whereas the darker spheres represent vesicles that are freely diffusing in the cytoplasm.

The sketch shown in Fig 1 shows the cytoplasm of chromaffin cells with neurosecretory vesicles immersed in and occasionally transported along microtubules (thick lines) or actin filaments (thin lines). In addition to its role as a barrier between the cytoplasm and the active sites [13, 14] the cortical actin network has an active role in exocytosis. It contributes to the transport of SVs in the periphery of the cell [15], facilitates the transport of SVs through the cellular cortex towards the sites of exocytosis and it also contributes mechanically to the exocytosis [16, 17].

Vesicles undergoing directed motion along microtubules often cover relatively large distances [18]. Some vesicles exhibit a restricted behaviour [19] termed “caged”. At the cell cortex (see Fig 1), this is generally associated with anchoring to the actin meshwork [12]. It has also been suggested that within this dense actin meshwork, secretory vesicles can become “functionally entrapped” and hence also display caged behaviour [18]. Not much is known about the specific mechanisms that may cause caged dynamics of SV in the central region of the cell. Note, however, that specifically the region close to the Golgi apparatus is abundant in actin concentration and that there is evidence for Golgi-associated actin (for a review see [20]).

In [19] live-cell imaging of nicotine stimulated bovine adrenal chromaffin cells was used to track the motion of secretory vesicles in 3-dimensions by confocal microscopy. Vesicles were categorised as undergoing either directed, free or caged motion and according to their distance from the plasma membrane. The measured transition rates implied that upon nicotine stimulation, cells spatially adjust their secretory vesicle pools. Specifically, it was reported that the fraction of vesicles undergoing directed transport increases as well as the rate by which vesicles enter directed transport. Both effects appear to contribute to the efficient replenishment of the pools of releasable vesicles. What feedback mechanisms, however, trigger those adjustments is not currently known.

The aim of this study is to introduce a quantitative description of secretory vesicle transport in chromaffin cells that is well adapted to the available data. Our goal is to show that spatial redistribution of vesicles upon stimulation can trigger the observed up-regulation of directed transported upon stimulation.

Modelling studies of intracellular transport have so far focused on transport along and within bundles of fibres [21–24]. On the other hand cytoplasmic vesicle transport as part of the secretory pathway has not received much attention from modellers so far: In [25] the biochemical reaction network underlying exocytosis has been modelled through a system of rate equations. Agent-based models have been considered in various studies such as in [26] which focuses on simulation methods, as well as in [27] on spatial aspects of vesicular sorting into different compartments and in [28] on aspects of pattern formation.

Mathematical modelling of the transport of secretory vesicles in chromaffin cells has so far been focusing on arrival and release statistics. Amperometric techniques make it possible to record the discrete arrival times of newly trafficked vesicles at the plasma membrane and hence provide insight into the underlying nature of the secretory pathway. In [29] it was shown that these arrival times follow a non-Poissonian probability distribution suggesting that there is an active recruitment process underlying the replenishment of releasable vesicles upon stimulation. More specifically, through the coupling of an attractive harmonic potential to a random process modelling vesicle migration, key aspects of arrival time statistics could be explained. Nevertheless, such a phenomenological model does not have the potential to link this effect to structural properties of the intracellular environment.

In order to address this issue, a recent study [30] suggested a system of advection-diffusion equations [31] as a tool to explore the various features of up-regulation of directed vesicle transport reported in [19]. It was found that spatial redistribution triggered by nicotine stimulation explains the observed bias toward *outward transport as compared to inward* transport upon stimulation. The parameter space for this class of models, however, turned out to be too high-dimensional prohibiting meaningful parameter fitting given the limited amount of available data [19]. As a consequence, this study could not address the question whether and under which additional assumptions the spatial redistribution that occurs upon stimulation can explain other more intricate aspects of up-regulation such as the much larger overall fraction of vesicles undergoing directed transport.

In our study we construct a conceptually simpler Markov state model to address these questions. This allows for a direct incorporation of the transition rates which are reported in [19] individually for each of the three spatial compartments: central, subcortical and peripheral.

We indeed find that the Markov chain based on the reported transition rates predicts spatial redistribution upon stimulation. Adding the additional assumption obtained through parameter fitting that the transport network in the central part of the cell has an outward bias, we find that this amplifies the overall fraction of vesicles undergoing directed transport in agreement with observations. We illustrate the underlying mechanism using an even simpler minimal Markov state model.

### Preliminary experimental results

**Fig 2:**
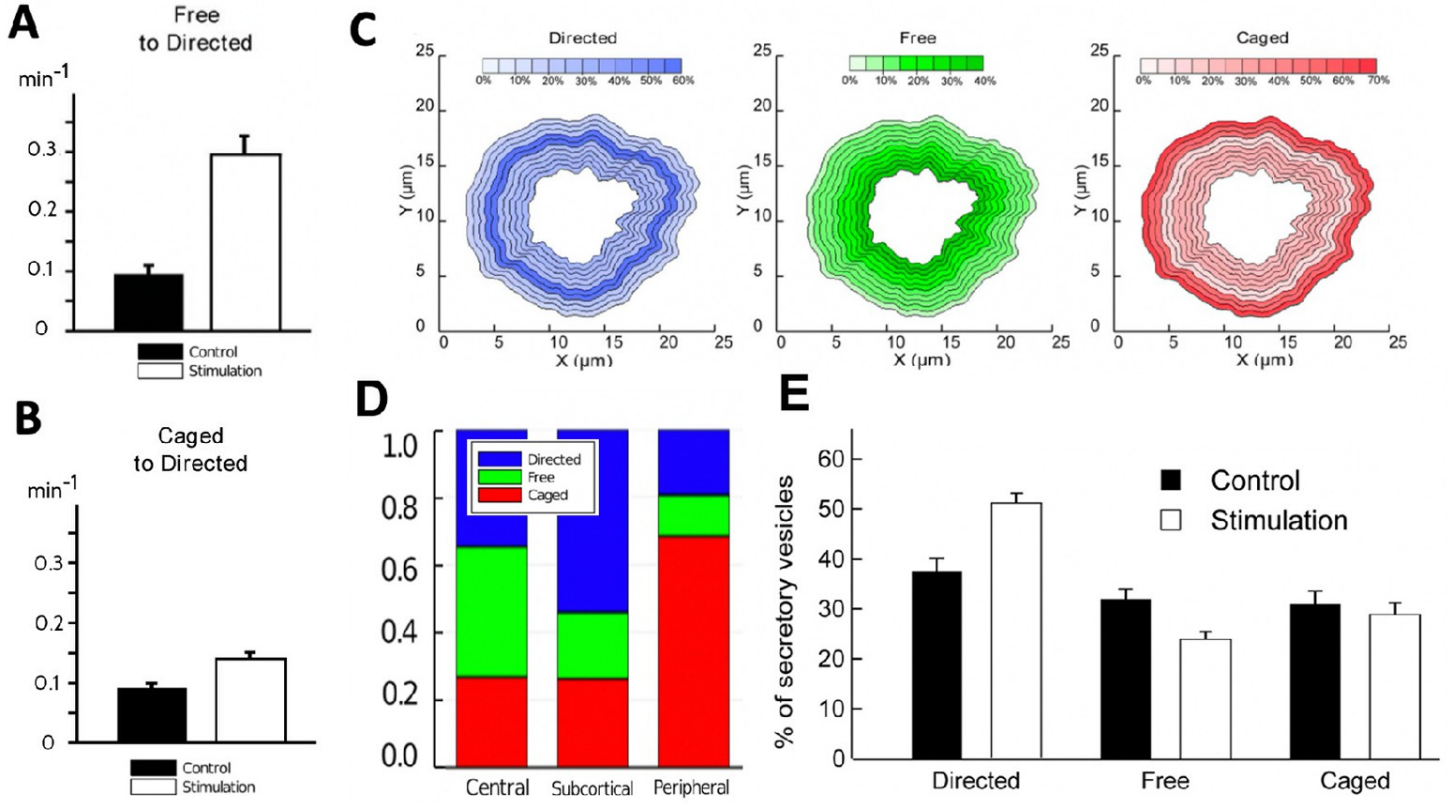
Statistics of tracked vesicles reported in [19]. (**A**) Fig 3H (with rates per 2 min) in [19]: average transition rates from free to directed motile state in control and stimulation. (**B**) Fig 3B (with rates per 2 min) in [19]: average transition rates from caged to directed motile state in control and stimulation. (**C**) Fig 2C in [19]: Proportions of vesicle motile behaviour in spatial bands of radial width 0.5μm. (**D**) Data in Fig 2C from [19] averaged within the three spatial compartments. (**E**) Fig 1E in [19]: Percentages of secretory vesicles in each motile state in control and stimulation.

**Fig 3:**
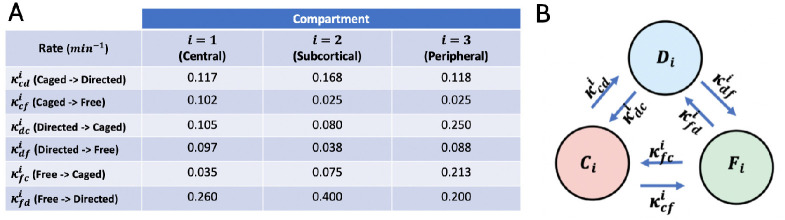
(**A**) Experimentally observed transition rates. Table of transition rates reported in [19]. (**B**) Graphical sketch of 3-state Markov chain model (*C*_*i*_(aged), *D*_*i*_(irected), *F*_*i*_(ree)) for single spatial compartments *i* = 1, 2, 3.

In [19] bovine adrenal chromaffin cells were imaged using time-lapse z-stack confocal imaging. A number of equatorial slices in the x-y plane of chromaffin cells were imaged so that 20% of the cell’s latitudinal range was covered. As a consequence, most of the secretory vesicle activity occurred within the imaged region. Individual vesicles were then tracked and the growth of each trajectory’s mean square displacement as a function of time—sub-linear, linear or supra-linear—was used to categorize them as either *caged*, *free* or *directed*. Comparing vesicles in control and in nicotine stimulated cells, the following key findings were reported:

1 *Up-regulation of directed transport*: Upon stimulation the fraction of vesicles undergoing directed motion grows from roughly 38% to 52% (Fig 1E in [19] which is reproduced in Fig 2E).
2 *Bias towards outward transport*: While vesicle transport is unbiased in control cells, in stimulated cells the ratio of outward vs. inward transport is roughly 2:1 (Fig 3S and 3T in [19]).

In addition to categorising vesicle trajectories, the transitions of vesicles from one motile state to another were recorded in trajectories spanning periods of 2 minutes. It was found that

3 stimulation leads to a *three-fold increase of the rate by which vesicles transition from free (unbiased random walk) motion to directed motion* (Fig 3H in [19] reproduced in Fig 2A).

Moreover, determining the transition rates within spatial bands of radial width 0.5 μm revealed three functionally distinct ring-shaped spatial compartments (visualised in Fig 1): the *peripheral* (0.5–1.5 μm distance from cell membrane), *subcortical* (1.5–2.5 μm) and *central* (2.5–5.0 μm). Note that when determining the average transition rates for these compartments we omit the outermost radial band right at the cortex which is mostly populated by caged vesicles. Fig 3 provides the transition rates reported in Fig 3J-3R in [19] (per 2 minutes) converted to numerical values per 1 minute as well as a sketch of the underlying 3-motile-states model.

They provide at least speculative insight into the underlying architecture of the intracellular transport network: The higher free-to-directed transition rate in the subcortical compartment suggests the presence of a higher density of cytoskeleton fibres in this region. Another observation is that the free-to-caged transition rate is significantly elevated in the peripheral region which might reflect a relatively higher density of the peripheral actin meshwork [32]. The higher caged-to-directed transition rate in the subcortical compartment as compared to the practically identical rates in the central and peripheral ones suggests that the microtubules and actin meshwork are both dense in this compartment or that they achieve significant overlap.

The directed-to-free transition rates are relatively higher in the central and peripheral compartments in comparison to the subcortical rate. We hypothesise that this is due to the higher number of microtubules terminating within these regions by which the faster off-rates there might also reflect the detachment of vesicles after reaching the “end of the track”. Another interesting observation from Fig 3 is that the caged-to-free transition rate is relatively higher in the central compartment, whereas free-to-caged rate is the lowest in the central compartment. This might be evidence that recently synthesised vesicles might enter the transport network as initially caged vesicles which is supported by the literature [20].

## Materials and methods

Our goal is to develop a Markov state model based on the transition rates listed in Fig 3. In [19] these have been published for stimulated cells, but not for control cells. We will therefore assume that the transition rates listed in Fig 3 in principle are valid irrespective of stimulation vs non-stimulation. Note that in the context of our model, this implies that stimulation does not alter the characteristics of how secretory vesicles behave in the cytoplasm, and especially interact with the cytoskeleton.

We will show that such a model is able to explain the key experimental observations 1. and 2. listed above. Spatial inhomogeneity of the cytoskeleton, specifically the non-uniform spatial distribution of outward vs inwards directed vesicle transport, coupled with the spatial redistribution of vesicles upon stimulation, will trigger up-regulation of outward transport and of overall transport as listed above. We refer to this mechanism as the “acceleration through spatial redistribution” hypothesis. We will start by introducing a minimal (”toy”) model in order to provide an intuitive initial illustration of the mechanism.

### Minimal 4-state model

**Fig 4:**
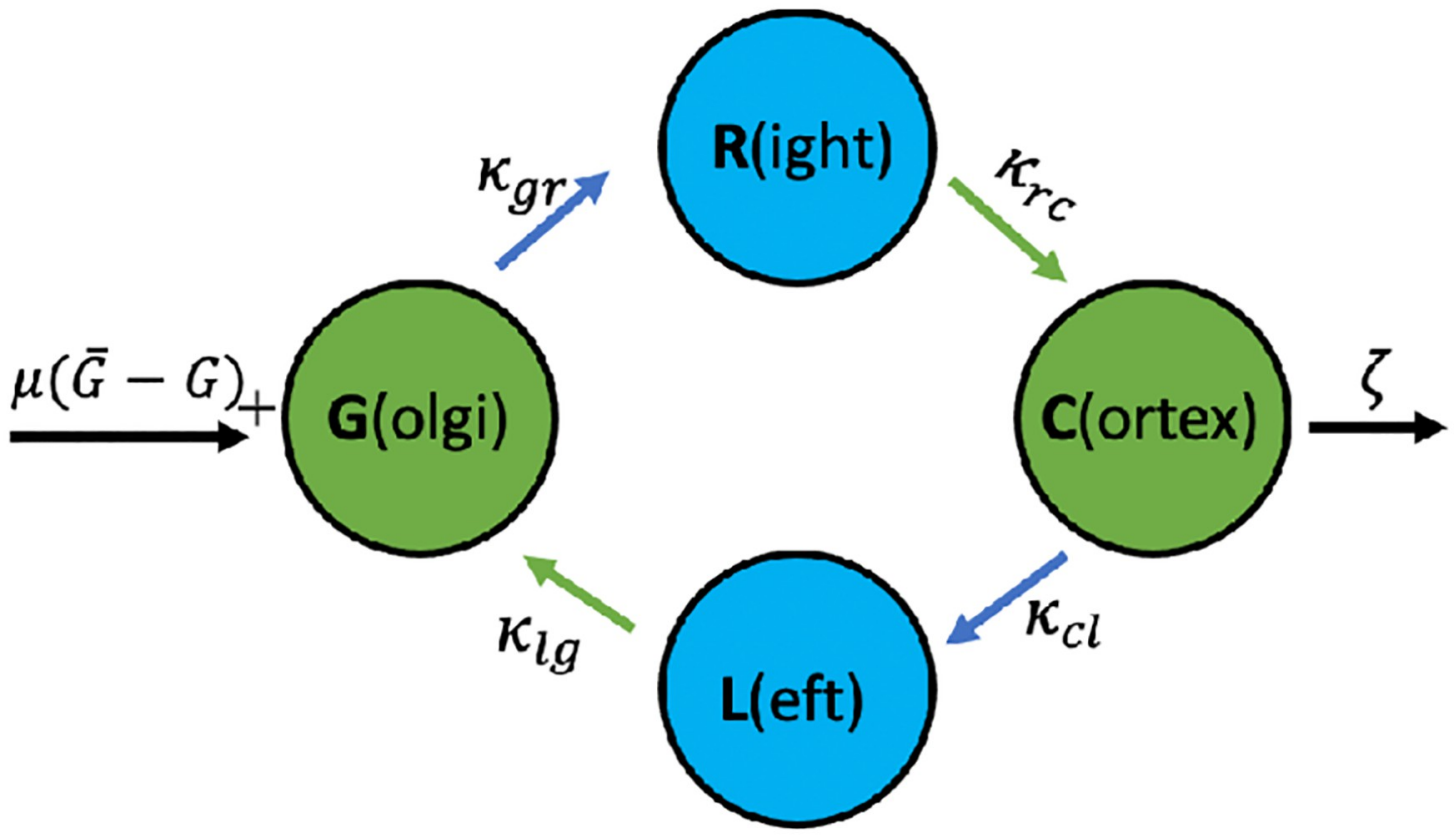
Graphical sketch of the minimal 4-state model.

The following 4-state continuous time Markov chain visualised in Fig 4 provides a proof of concept for the “acceleration through spatial redistribution” hypothesis.

In this simple model vesicles exist in one of four states, respectively spatial compartments: They are generated at the Golgi apparatus (G), from where they might engage in outward—visualised as right-directed—transport (R) towards the cell cortex (C). Vesicles at the cortex engage in in-ward—shown as left-directed (L)—transport and ultimately arrive back at the Golgi. Alternatively, vesicle at the cortex may enter the pathway towards exocytosis with rate *ζ*. This pathway acts as a sink whereas the cell’s synthesis of vesicles near the Golgi represents a source of new secretory vesicles. We assume that the release of vesicle from the trans-Golgi network is regulated by feedback mechanisms which aim at maintaining a given number of $\bar{G}$ vesicles at the Golgi apparatus.

This gives rise to the system of rate equations

$$\begin{array}{c} \begin{array}{cc} \dot{G}\left(t\right) & =\mu {\left(\bar{G}-G\right)}_{+}-\kappa_{gr}\;G\left(t\right)+\kappa_{lg}\;L\left(t\right)\;,\\ \dot{C}\left(t\right) & =\kappa_{rc}\;R\left(t\right)-\kappa_{cl}\;C\left(t\right)-\zeta \;C\left(t\right)\;,\\ \dot{R}\left(t\right) & =\kappa_{gr}\;G\left(t\right)-\kappa_{rc}\;R\left(t\right)\;,\\ \dot{L}\left(t\right) & =\kappa_{cl}\;C\left(t\right)-\kappa_{lg}\;L\left(t\right)\;, \end{array} \end{array}$$ (1)

where the subscript _+_ denotes the positive part of the expression and reflects the assumption that vesicles at the Golgi apparatus may be released into the cytoplasm but not removed. In order to verify the up-regulation through spatial redistribution hypothesis, we take *ζ* > 0 as close to zero in a control cell and assume that stimulated cells are characterised by significantly larger *ζ*. In our assessment we only consider the steady state solution (for details see supplementary section S1 Steady state solution of the minimal 4-state model in S1 File) where *μ* is large, i.e. where $G=\bar{G}$ and

$$\begin{array}{c} C=\frac{\kappa_{gr}}{\zeta +\kappa_{cl}}G\;,\;R=\frac{\kappa_{gr}}{\kappa_{rc}}G\;,\;L=\frac{\kappa_{gr}\kappa_{cl}}{\kappa_{lg}\left(\zeta +\kappa_{cl}\right)}G\;. \end{array}$$ (2)

In the context of this minimal model up-regulation of outward transport is characterised by how $\frac{R}{L}$ depends on *ζ*, namely

$$\begin{array}{c} \frac{R}{L}=\frac{\kappa_{lg}}{\kappa_{rc}}(1+\frac{\zeta }{\kappa_{cl}})\;, \end{array}$$ (3)

which increases monotonically as a function of *ζ* since all transition rates are positive. This shows that the minimal model predicts a bias towards outward transport in response to stimulation. Note that the decrease of the number of vesicles in *L* is triggered by a preceding decrease in the compartment C (see (2)). In this sense the observed up-regulation is a consequence of the adjustment of the spatial distribution upon stimulation.

Note that to mimic a ratio of *R*: *L* ∼ 1 which is observed in control cells (*ζ* small) the rates *κ*_*lg*_ and *κ*_*rc*_, which both represent off-rates from vesicle transport, are required to be approximately equal as a consequence of (3).

Up-regulation of directed transport upon stimulation, on the other hand, is characterised by the ratio $\frac{R+L}{G+C}$, which—at steady state—is given by

$$\begin{array}{c} \frac{R+L}{G+C}=\frac{\kappa_{gr}}{\kappa_{rc}}(1+\frac{1}{\kappa_{lg}}\frac{\alpha \gamma -\beta \nu }{\alpha +\zeta })\;, \end{array}$$ (4)

where *ν* = *κ*_*gr*_ − *κ*_*cl*_, $\beta =\frac{1}{2}\left(\kappa_{rc}+\kappa_{lg}\right)$, *α* = *κ*_*gr*_ + *κ*_*cl*_, $\gamma =\frac{1}{2}\left(\kappa_{rc}-\kappa_{lg}\right)$. Note that *α* and *β* are both positive. Therefore, the minimal model predicts up-regulation of directed transport only if *ν* > 0 and/or *γ* < 0. The fact that we need *κ*_*rc*_ ∼ *κ*_*lg*_ for an 1:1 ratio of right and left moving vesicles in control suggests *γ* ∼ 0 and therefore we require *ν* > 0.

Interpreting the on-rates *κ*_*gr*_ and *κ*_*cl*_ as representatives of effective fibre densities—the more cytoskeleton fibres and motor proteins moving in the right direction are available, the larger the respective on-rates—the result that *ν* > 0 indicates that there should be a higher net density of fibres triggering outward transport as compared to fibres resulting in transport away from the cortex. It is worth noticing that small *κ*_*cl*_ favours both, up-regulation of directed transport in (4) and a strong bias towards outward transport (*R*/*L*, see (3)) upon stimulation.

Finally, it is important to note that there is an inherent asymmetry in this minimal model. Specifically, only transitions from the spatially interior state G to right directed motion are considered and not vice versa. Similarly, we only consider transitions from the spatially outward state C to left directed motion and not vice versa. This restriction makes sense for a simplified model thought to be coarse-graining a more intricate underlying system. In the following development of the full Markov state model we consider three spatial compartments with bi-directional transitions modelling vesicle attachment and detachment from cytoskeleton fibres and molecular motors. The asymmetry of the minimal model will be replaced by transitions between spatial compartments modelling directed transport.

### Markov state model

Our goal is to formulate a mathematical model which explains the key experimental observations reported in [19]. To this end we refine the minimal model visualised in Fig 4 by adding spatial compartments building on the measured rates listed in Fig 3.

We represent the spatial distribution of vesicles by assigning them to one of the three compartments *central* (i = 1), *subcortical* (i = 2) and *peripheral* (i = 3), which mimics the terminology used in [19].

**Fig 5:**
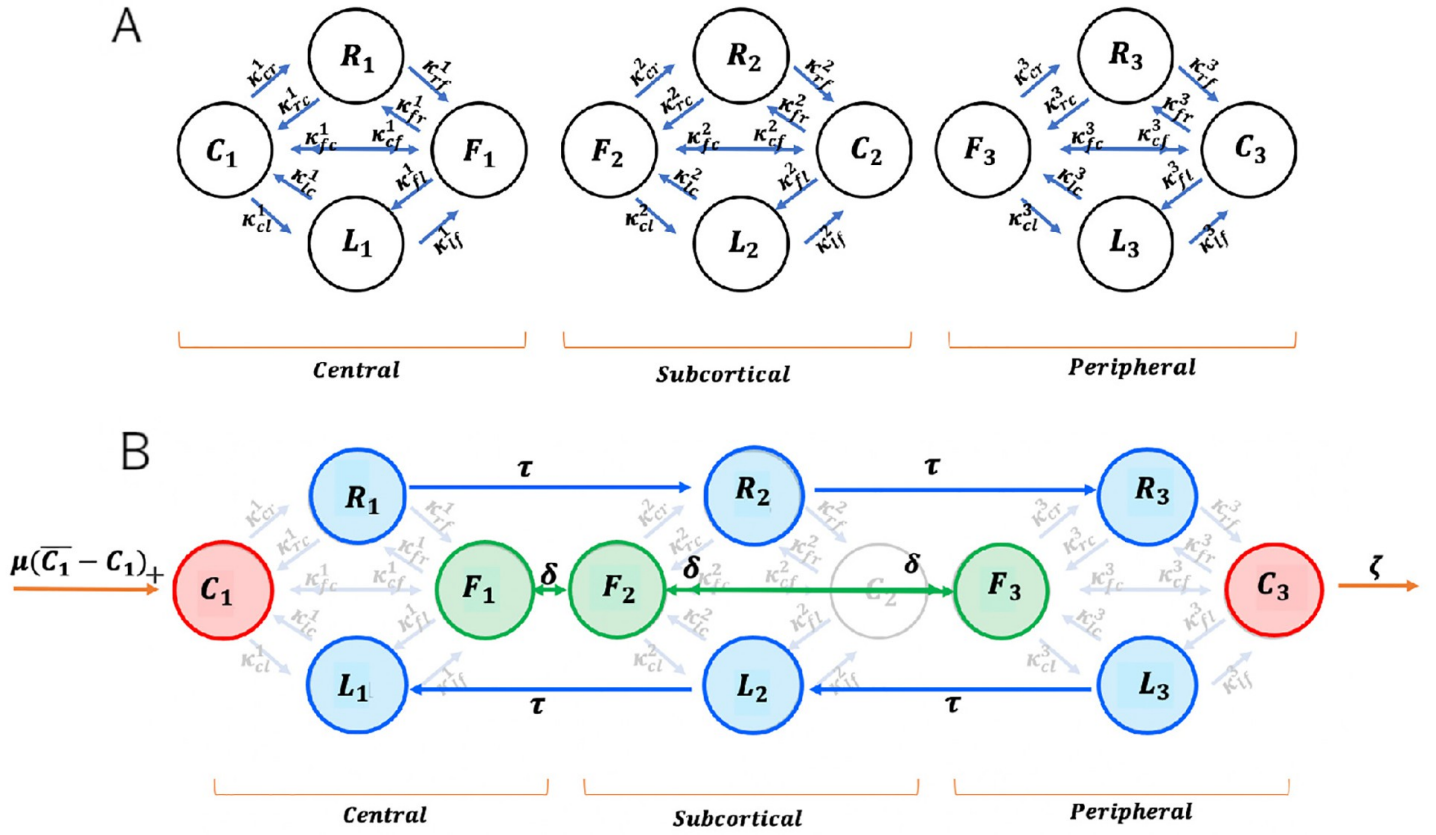
Visualisation of Markov states with intra-compartmental transitions (A) and inter-compartmental transitions (B).

In each spatial compartment vesicles are either in state *F*_*i*_ (diffusing *freely* in the cytoplasm), *C*_*i*_ (*caged*, i.e. undergoing random, though spatially restricted motion) or undergoing directed motion in state *R*_*i*_ (towards the cell periphery usually visualised on the *right*) or *L*_*i*_ (towards the cell centre usually visualised on the *left*). Note that in the context of the minimal model visualised in Fig 4 the state **C** stands for cortex and corresponds approximately to the union of the states *C*_3_ and *F*_3_ in the detailed model Fig 5, whereas the state **G**(olgi) of the minimal model approximately represents the union of *C*_1_ and *F*_1_.

Fig 5A provides a visualisation of the transitions occurring within the individual spatial compartments of the model. Note that the experimental study only provides information about the rates by which either free or caged vesicles engage in directed transport, but not about the direction of transport (Fig 3).

For this reason we introduce the directional parameter 0 ≤ *ρ*_*i*_ ≤ 1 for each of the three spatial compartments *i* = 1, 2, 3. The role of this parameter is to determine how the experimentally recorded on-rates into directed transport $\kappa_{fd}^{i}$ and $\kappa_{cd}^{i}$ from Fig 3 distribute into outward (right) and inward (left) transport. Using the directional parameters we write the transition rates from the free and caged states into either left or right transport shown in Fig 5A as

$$\begin{array}{c} \begin{array}{cc} \kappa_{fr}^{i} & =\rho_{i}\;\kappa_{fd}^{i}\;,\\ \kappa_{fl}^{i} & =\left(1-\rho_{i}\right)\;\kappa_{fd}^{i}\;, \end{array}\;\begin{array}{cc} \kappa_{cr}^{i} & =\rho_{i}\;\kappa_{cd}^{i}\;,\\ \kappa_{cl}^{i} & =\left(1-\rho_{i}\right)\;\kappa_{cd}^{i}\;. \end{array} \end{array}$$ (5)

Here we use the overbar notation to indicate that these rates will be further modified below. One way of interpreting the factors *ρ*_*i*_ is as the fraction of fibres within one of the spatial compartments along which vesicles will be transported outwards vs inwards. This implies that *ρ*_*i*_ does not reflect the concentration of fibres within the respective spatial compartment. Instead the rates $\kappa_{fd}^{i}$ are likely to reflect the net fibre densities in any given compartment.

Similar considerations for off-rates are not necessary, so we assume that

$$\begin{array}{c} \kappa_{rf}^{i}=\kappa_{lf}^{i}=\kappa_{df}^{i}\;\text{and}\;\kappa_{rc}^{i}=\kappa_{lc}^{i}=\kappa_{dc}^{i} \end{array}$$ (6)

for all three spatial compartments *i* = 1, 2, 3, i.e. the experimentally observed off-rates from directed transport reported in Fig 3 taken from [19] are used irrespective of the direction of transport.

In order to connect the so-far isolated spatial compartments sketched in Fig 5A, we introduce additional transitions accounting for the random and directed motion of vesicles. The mean square displacement per time of free vesicle trajectories in 3D has been determined in [19] (S1 Fig in S1 File) as 0.75 μm^2^/min. Assuming an average distance of 1.7μm between our three, linearly aligned, spatial compartments this suggests a inter-compartment transition rate of *δ* = (0.75/6)/1.7^2^ min^−1^ ≈ 0.04 min^−1^ to account for random motion of free vesicles. The corresponding transitions between the states of free vesicles in neighbouring compartments are visualised in Fig 5B as bidirectional arrows.

We also include uni-directional transitions between the directed states in order to represent directed transport of secretory vesicles. In Fig 5B these are shown as blue arrows. Instantaneous maximal vesicle speeds of up to 0.05 μm s^−1^ have been reported in [19]. This would suggest transition rates of 0.05 × 60/1.7 ≈ 1.8 min^−1^. In steady state solutions of the Markov state model, however, these fast transition rates would result in vesicles pooling at the states *R*_3_ and *L*_1_ which is not supported by observations [19]. Instead, we assume that vesicles achieve maximal speeds only intermittently and estimate the transition rate of vesicles undergoing directed transport as *τ* = 0.15 min^−1^.

Additional transitions embed the Markov State model (visualised in Fig 5B) within the life-cycle of secretory vesicles. We model the release of newly synthesised vesicles at the trans-Golgi network apparatus as an in-flux of vesicles into *C*_1_ (the caged pool close to the Golgi apparatus) with rate $\mu {\left({\bar{C}}_{1}-C_{1}\right)}_{+}$. Here, too, we use the subscript _+_ to restrict this expression to positive values returning zero in case $({\bar{C}}_{1}-C_{1})<0$. At steady state for *ζ* > 0 this term effectively imposes the constant value ${\bar{C}}_{1}$ on the number of vesicles in *C*_1_. For our purposes *μ* is an arbitrary large value which has no effect on the steady state solutions and ${\bar{C}}_{1}$ is an arbitrary constant which factors out of the steady state solution after we normalise either by the total number of vesicles in the system or in the respective spatial compartment. Having vesicles enter the system through the node *C*_1_ as opposed to the central node of free vesicles *F*_1_ is speculative and reflects evidence reviewed by [20] that Golgi-associated actin is involved in fission of transport vesicles from the *trans*-Golgi network.

Finally, as in the minimal 4 state model, we model the onset of the pathway through which vesicles undergo exocytosis by an outgoing-transition (rate) from the pool of caged vesicles in compartment *i* = 3 (peripheral) denoted by *ζ*. This reflects the role the actin cortex plays in the exocytosis pathway [32]. With these parameter values, especially with the estimated values for *τ* and *ζ* steady state simulations of stimulated cells exhibit relative shares of vesicles (free, caged and undergoing directed motion) Fig 7A which coincide well with measured values from [19] visualised in Fig 2C and 2D. Note that the full system of rate equations corresponding to the Markov chain shown in Fig 5 is shown in supplementary section S2 Governing Equations in S1 File.

### Fitting of directional parameters

We determine the directional parameters *ρ*_1_, *ρ*_2_ and *ρ*_3_ (see Table 1) by fitting (least-squares) the predicted ratios of right vs left moving vesicles denoted by Φ_*ζ*_ and the predicted ratio of directed motion vs random motion denoted by Ψ_*ζ*_. These quantities are given by

$$\begin{array}{c} \Phi_{\zeta }=\frac{\sum_{i=1}^{3}\left(R_{i}^{\zeta }+L_{i}^{\zeta }\right)}{\sum_{i=1}^{3}\left(F_{i}^{\zeta }+C_{i}^{\zeta }\right)}\;\text{and}\;\Psi_{\zeta }=\frac{\sum_{i=1}^{3}R_{i}^{\zeta }}{\sum_{i=1}^{3}L_{i}^{\zeta }}\;, \end{array}$$

where i is the spatial compartment index and $R_{i}^{\zeta }$, $L_{i}^{\zeta }$, $F_{i}^{\zeta }$ and $C_{i}^{\zeta }$ are the steady state abundances for a given exocytosis rate *ζ*.

**Table 1:** Parameter values.

Description	Symbol	Value	Reference
Inter-compartmental transition of free vesicles	*δ*	0.04 min^−1^	S1 Fig in [19].
Transition into exocytosis pathway (control)	*ζ*_ctr_	10^−4^ min^−1^	Estimated as a small value arbitrarily close to 0.
Transition into exocytosis pathway (stimulated cells)	*ζ*_stim_	0.15 min^−1^	Estimated to reproduce the relative shares of motile states in Fig 2C and 2D.
Directed spatial transition	*τ*	0.15 min^−1^	Estimate limited by maximal instantaneous speed (Table 1 in [19]).
Outward vs inward transport	*ρ*_1_, *ρ*_2_, *ρ*_3_	0.75, 0.6, 0.4	Estimated (see section Fitting of directional parameters)

We take into account the deviations of those quantities from the observed ones for both, stimulated cells and control cells. The error functional which we minimise is therefore given by

$$\begin{array}{c} E={\left(\Phi_{\zeta_{\text{stim}}}-\Phi_{\text{stim}}^{\text{obs}}\right)}^{2}+{\left(\Phi_{\zeta_{\text{ctr}}}-\Phi_{\text{ctr}}^{\text{obs}}\right)}^{2}+{\left(\Psi_{\zeta_{\text{stim}}}-\Psi_{\text{stim}}^{\text{obs}}\right)}^{2}+{\left(\Psi_{\zeta_{\text{ctr}}}-\Psi_{\text{ctr}}^{\text{obs}}\right)}^{2}\;, \end{array}$$ (7)

where the experimentally observed values are given by $\Phi_{\text{ctr}}^{\text{obs}}=0.38/0.62\approx 0.6$, $\Phi_{\text{stimu}}^{\text{obs}}=0.52/0.48\approx 1.1$ (see Fig 2E) as well as $\Psi_{\text{ctr}}^{\text{obs}}=1.0$, $\Psi_{\text{stimu}}^{\text{obs}}=2.0$.

### Numerical computations

We used the open-source software Julia [33] for numerical computations. The software package *Catalyst* was used to formulate the model. Steady state solutions have been computed as long time solutions of the respective dynamic models: The package *DifferentialEquations* was used to compute solutions of systems of ODEs and we used the package *DiffEqJump* for stochastic simulations. The package *Optim* was used for optimisation.

## Results

### Stimulated exocytosis triggers spatial rearrangement of chromaffin vesicles

We numerically compute the steady state solutions of the Markov state model visualised in Fig 5 with the rates (5) and (6).

**Fig 6:**
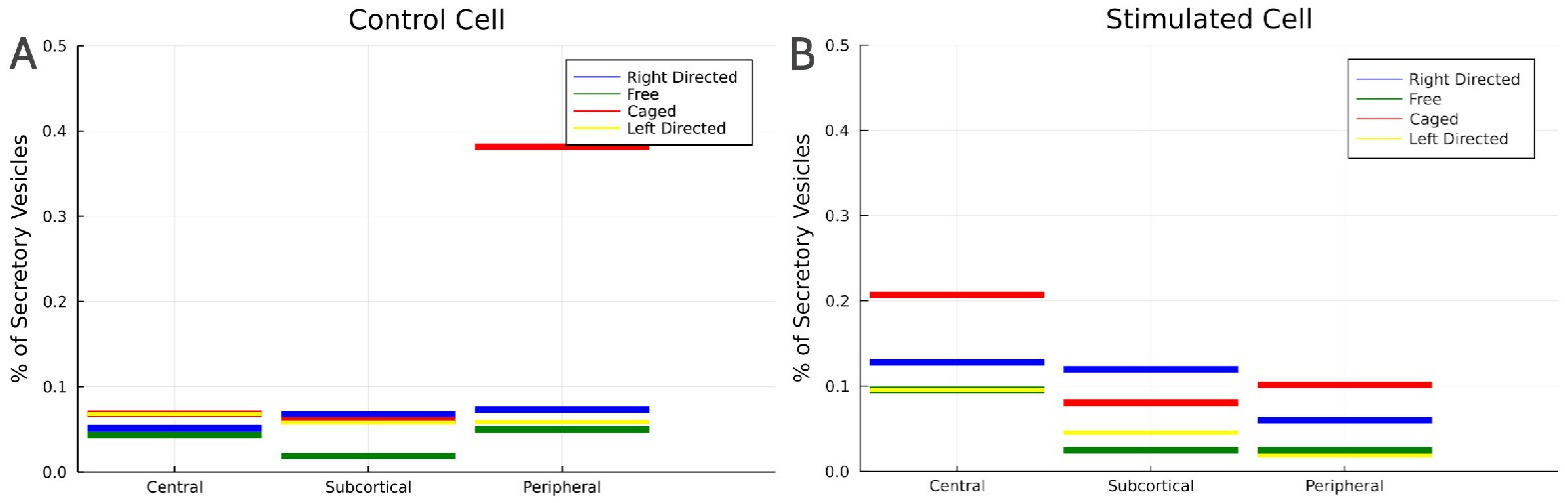
Simulated steady state probability distributions of vesicles in control (A) and upon stimulation (B), both normalised so they admit a probabilistic interpretation.

In simulated control cells, the steady state distribution of vesicles (Fig 6A) is characterised by a large portion of vesicles (of about 38%) being pooled in the caged state at the cortex. In the corresponding steady state distribution for a stimulated cell (Fig 6), this pool of vesicles ready to enter the pathway leading to exocytosis is largely reduced. This results in caged vesicles being distributed more evenly between the three spatial compartments upon stimulation with most of them now being located in the central region of the cell. All together this accounts for a significant shift in the spatial distribution of vesicles from the cell periphery to the cell centre as a consequence of the permanent out-flux of vesicles at the cortex in response to secretagogue stimulation. This is the effect termed “redistribution” in this study. It needs to be noted, however, that this terminology is somewhat inaccurate as it indicates that vesicles are merely spatially relocated. Instead, upon stimulation the pool of vesicles undergoes permanent turnover and the steady state simulation results before normalisation (S1 Fig in S1 File) indicate that upon stimulation their abundance decreases.

**Fig 7:**
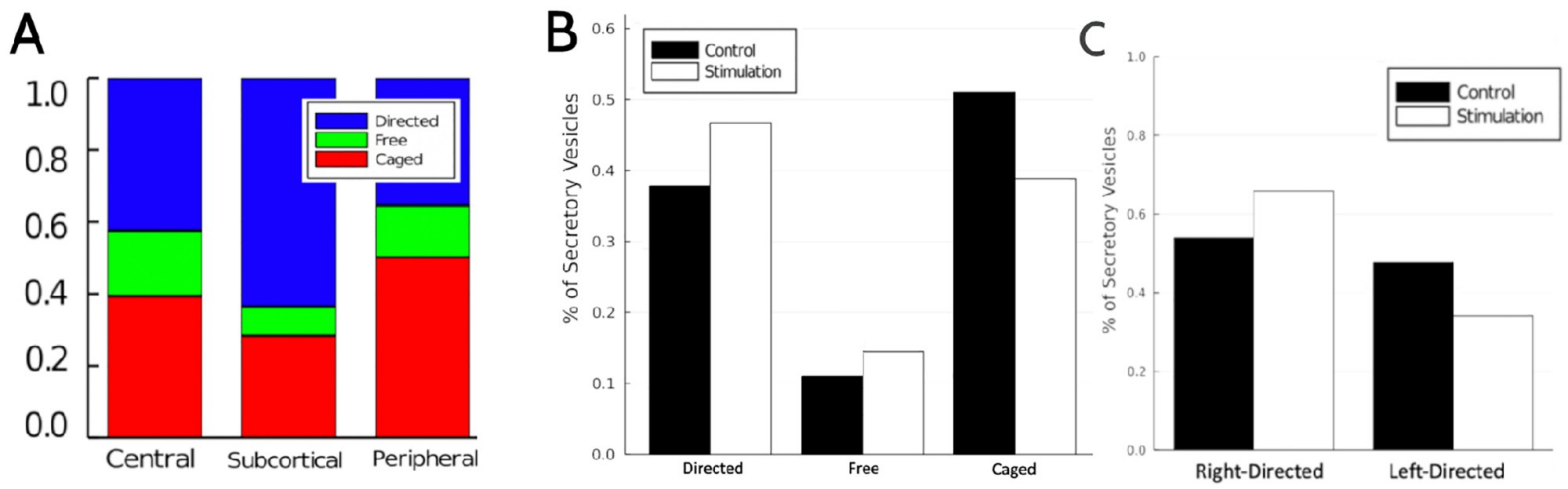
Steady state simulation results: **A** Intra-compartmental proportions in stimulated cells. **B** Percentages of secretory vesicles in each motile state. **C** Percentages of secretory vesicles undergoing direction motion (right vs left).

From the results of Fig 6 we extract the intra-compartmental proportions for a stimulated cell which are visualised in Fig 7A and qualitatively agree with the experimentally recorded intra-compartmental proportions shown Fig 2C and 2D.

### Spatial redistribution can explain both, up-regulation of directed transport and bias towards the plasma membrane

Our model predicts an increase from 38% to 47% in the share of vesicles undergoing directed transport in stimulated cells vs control (Fig 7B). This compares well with the experimental data reproduced in Fig 2E. Likewise the rate of outward moving vesicles vs. inward moving vesicles increases from about 1:1 in control to almost 2:1 in stimulated cells (Fig 7C) which also coincides with the data reported in [19].

The vesicle distributions in Fig 6 reveal that upon stimulation the relative abundance of vesicles moving outwards (blue) increases in the central and subcortical compartment. On the other hand the relative abundance of vesicles moving inwards (yellow) decreases in the peripheral compartment. Both effects contribute to the up-regulation of total and outward directed transport. They are both triggered by spatial redistribution of vesicles upon stimulation since the states in which most of the redistribution takes place are those which precede states of direct motion: from *C*_1_ and *F*_1_ vesicles transition into *R*_1_—and later *R*_2_ and *R*_3_, from *C*_3_ they transition into *L*_3_—and later back into *L*_2_ and *L*_1_.

This mechanism is overshadowed by the dense network of potential transitions in the Markov State model Fig 5 and so it is easier to understand it in the context of the minimal model visualised in Fig 4. This simpler model also features redistribution of non-moving vesicles upon stimulation, namely through the number of vesicles in state C(ortex) which decreases upon stimulation (*ζ* large) according to Eq (2). As a consequence, upon stimulation the number of vesicles in the state L(eft) also decreases, since C is the only state from which vesicles transition into L. This readily explains the increase of the ratio R:L under stimulation (see Eq (3)) in the minimal model. It also indicates that in the full Markov state model the mechanism which drives the increase of the ratio of outward vs inward transport upon stimulation is the redistribution of caged and free vesicles upon stimulation.

The mechanism which drives the up-regulation of the total share of vesicles undergoing directed transport is more obscure. For the minimal model we have concluded that up-regulation of total directed transport requires *ν* = *κ*_*gr*_ − *κ*_*cl*_ > 0 in Eq (4) (or alternatively *γ* < 0 which we ruled out assuming that 2*γ* = *κ*_*rc*_ − *κ*_*lg*_ = 0). In other words, the rate by which vesicles enter the state representing transport to the R(ight) should be faster than the corresponding rate by which vesicles enter the state L(eft). Again the underlying mechanism is triggered by the redistribution of non-moving cells upon stimulation which decreases the number of vesicles in C(ortex). If the rates, *κ*_*gr*_ and *κ*_*cl*_, were equal (i.e. *ν* = 0), then the ratio of L:C (Eq 10) and R:G (Eq 9) would be same before and after stimulation prohibiting upregulation of total transport represented by (*R* + *L*)/(*G* + *C*) (Eq (4)). However, with the rate to enter L being slower than the one to enter R, the decrease in C has a relatively smaller effect on R+L than on G+C and the share of vesicles undergoing directed transport increases.

### Outward directed structural bias of the central transport network promotes overall up-regulation of directed transport upon stimulation

**Fig 8:**
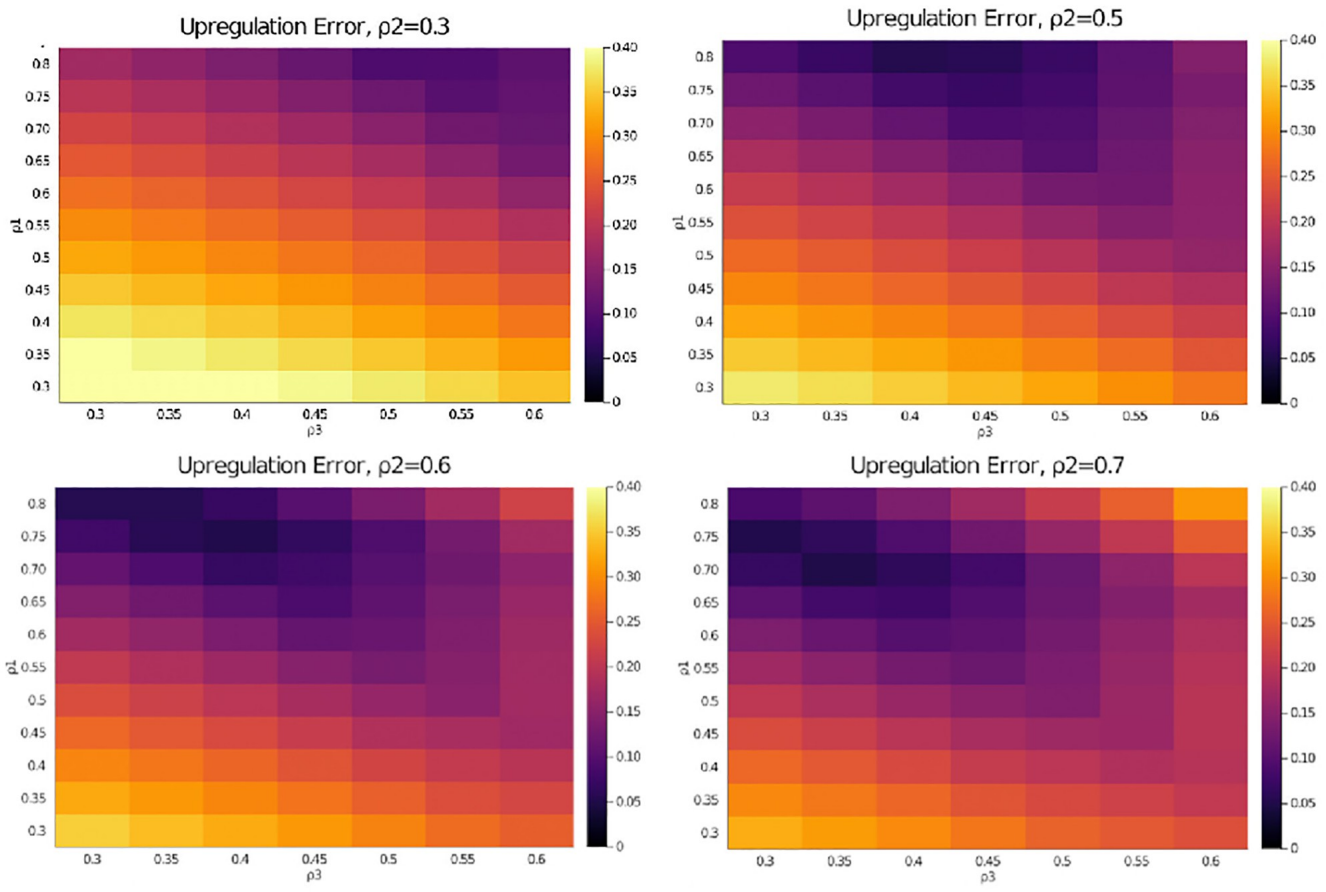
Heatmap plots of the predictive error (7) with respect to up-regulation of total and right vs left transport for different values of the directional parameters *ρ*_1_, *ρ*_2_ and *ρ*_3_.

Least squares fitting of the three directional parameters *ρ*_1_, *ρ*_2_ and *ρ*_3_ (Fig 8) by minimising the error functional (7) shows that in order for the model to correctly predict the observed up-regulation of both, outward and total directed transport, the directional parameters *ρ*_1_ and/or *ρ*_2_ are required to have an outward bias (>0.6). This observation is reminiscent of the requirement that *κ*_*gr*_ > *κ*_*cl*_ in the minimal model. It indicates that in the central and sup-peripheral part of the cell the vesicles’ propensity to engage in outward directed transport is higher than to engage in in-ward transport.

This is reminiscent of the observation that in many cell types microtubule minus ends are anchored at microtubule organising centres close to the nucleus favouring outward transport through molecular motors of the kinesin family [5]. In addition, microtubules are also nucleated at the Golgi with their minus ends remaining close to it [34, 35].

Nevertheless, note that the parameters *ρ*_1_, *ρ*_2_ and *ρ*_3_ are phenomenological. They do not indicate whether this effect is caused by the density of outward-transport fibres in the central and subcortical regions being relatively higher than the density of inward-transport fibres, or—alternatively—by faster rates of attachment to outward directed fibre-motor combinations.

What we conclude is that an intracellular transport network with a bias towards outward transport in the central and subcortical regions will trigger an up-regulation of total and outward vesicle transport in response to their spatial redistribution at stimulation.

## Discussion

In this study we formulate a Markov transition state model which is closely aligned to the data obtained from tracking secretory vesicles in chromaffin cells reported in [19].

We model the asymmetry of directed transport due to the topological constraints of the intracellular space: directed transport originating in the central compartment is biased towards the cell periphery, transport of vesicles originating in the peripheral compartment is towards the cell centre. In the Markov state model Fig 5B this is implemented by the central compartment having only a single transport transition (*R*_1_ → *R*_2_) directed towards the periphery, and by the peripheral compartment having only one transport transition *L*_3_ → *L*_2_ directed towards the cell centre.

Steady state simulations of control and stimulated cells show that secretagogue stimulation shifts the spatial distribution of secretory vesicles from the cell periphery towards the central region of the cell. The spatial redistribution upon stimulation leads to a significant increase of the overall share of vesicles undergoing outward transport. This agrees with observations [19] and with an earlier modelling study [30].

In addition our study suggests that the spatial redistribution also triggers the up-regulation of overall directed transport, i.e. irrespective of direction, observed in [19]. The mechanism relies on the (on-)rates by which vesicles enter the states representing outward transport being faster than the corresponding (on-)rates by which vesicles enter the states associated to inward transport. (Alternatively the (off-)rates by which vesicles leave the states representing outward transport could also be slower than the corresponding rates by which vesicles leave the inward transport states). In this study we illustrate this mechanism using the minimal model visualised in Fig 4.

For the full Markov state model Fig 5 parameter fitting shows that the required asymmetry originates from the central (and—to a lesser extent—the subcortical) compartment having a bias towards outward transport (*ρ*_1_ = 0.75 > 0.5, see Table 1). This indicates that the up-regulation at stimulation is an in-built property of the cell’s cytoskeleton architecture—fibre-motor assemblies in the central region of the cell having a bias towards outward transport—and not necessarily triggered by molecular feedback mechanisms.

## Conclusion

In this study we show that a mathematical model based on experimentally observed transition rates [19] between motile states of secretory vesicles in chromaffin cells predicts a spatial redistribution of vesicles and up-regulation of outward transport in response to secretagogue stimulation.

We show that this spatial redistribution combined with spatial inhomogeneity of the cellular transport network—a bias towards outward transport in the central region of the cell—predicts that upon stimulation a relatively larger share of vesicles undergoes directed transport in agreement with experimental data. This indicates that the up-regulation at stimulation is an in-built property of the cell’s cytoskeleton architecture, and not necessarily triggered by molecular feedback mechanisms.

We cannot, however, out-rule that biochemical regulations are involved in the process. This study confirms that an unregulated model could explain the observed up-regulation of directed transport. Regulations might therefore control additive elements to this inherent process. In this regard it is worth noting that only a few molecular motors are known to act in the neuroexocytic pathway. These include: (i) Myosin VI short insert which underpins the recruitment of SV to the cortical actin network in response to stimulation [12]; (ii) Myosin II which promotes the synchronized translocation of actin-bound vesicles to the plasma membrane [11]. Both these myosins act on the distal caging of SVs and not on the transport component. (iii) Myosin V which is the only processive Myosin involved in the mobility of secretory vesicles. Considering the orientation of the cortical actin network abutting the plasma membrane, however, it appears debatable whether it acts in directing vesicles towards the plasma membrane.

We therefore think that it is worth considering that the relatively simple model put forward in this study can explain this tantalizing transport mechanism which are often assumed to require molecular motors for which there are little evidence in the case of neuroexocytosis.

The mathematical model makes a series of testable predictions. While it does not appear feasible to alternate the structure of the cytoskeleton in a way which changes the directional bias of the transport network, manipulation of either plus end or minus end directed molecular motors involved in vesicle transport could have a similar effect. Our model predicts that if the cellular transport network has an inward bias, then the fraction of vesicles undergoing directed transport will be reduced in stimulated cells.

What could be simpler is to limit the rate by which vesicles in stimulated cells progress through the cortical actomyosin network to the site of exocytosis, e.g. through cytoskeleton drugs or genetic manipulation. In this case the Markov state model predicts that the up-regulation of both total and outward transport will be limited as a consequence of reduced spatial redistribution.

In [19] it was also reported that transition rates from the pool of free vesicles into directed transport accelerate three-fold upon stimulation (Fig 2A). Spatial redistribution alone cannot account for this since the slowest—among all spatial compartments—reported transition rate $\kappa_{fd}^{i}$ in stimulation is 0.2 min^−1^ (Fig 3), whereas the average rate reported in control is 0.1 min^−1^ (Fig 2A, which reports rates per 2 min). In the supplementary material section S4 Modification of the model introducing crowding in S1 File we present a modification of our model based on the assumption that specifically the central cytoskeleton network in control cells is limited by its carrying capacity. We show that this hypothetical mechanism is consistent with the observations (Fig 2A and 2B). It should be noted, however, that this is purely speculative and only meant to demonstrate the flexibility of the modelling approach. Indeed we believe that more experimental data would be required to draw a definitive conclusion about the underlying mechanism.

One limitation of this study is that it does not consider the details of the pathway by which the actomyosin cortex recruits secretory vesicles and supports their progress towards the active sites. Future research will address this question. Indeed not much is known about the regulation mechanisms that couple cytoplasmic transport of chromaffin vesicles and the regulation of exocytosis through the actin cytoskeleton [11, 18]. We believe that dynamic solutions of mathematical models such as the one introduced in this study have the potential to give insight into those regulation mechanisms, especially when combined with data on vesicle release statistics. Further, they will provide insights for futures studies on other trafficking events including that of autophagosomes [36] and synaptic vesicles in neurons [37].

## Supporting information

S1 File (PDF)

## References

[1] Gimenez-Molina Y, Villanueva J, Francés MdM, Viniegra S, Gutiérrez LM. Multiple Mechanisms Driving F-actin-Dependent Transport of Organelles to and From Secretory Sites in Bovine Chromaffin Cells. Frontiers in Cellular Neuroscience. 2018;12:344. doi: 10.3389/fncel.2018.00344 30356839 PMC6190647

[2] McMurray CT. Neurodegeneration: diseases of the cytoskeleton? Cell Death Differ. 2000;7(10):861–865. doi: 10.1038/sj.cdd.4400764 11279530

[3] Wang T, Martin S, Nguyen TH, Harper CB, Gormal RS, Martínez-Mármol R, et al. Flux of signalling endosomes undergoing axonal retrograde transport is encoded by presynaptic activity and TrkB. Nature Communications. 2016;7. doi: 10.1038/ncomms13768 PMC5427517 27687129

[4] Barlan K, Gelfand VI. Microtubule-Based Transport and the Distribution, Tethering, and Organization of Organelles. Cold Spring Harbor Perspectives in Biology. 2017;9(5). doi: 10.1101/cshperspect.a025817 28461574 PMC5411697

[5] Caviston JP, Holzbaur ELF. Microtubule motors at the intersection of trafficking and transport. Trends in Cell Biology. 2006;16(10):530—537. doi: 10.1016/j.tcb.2006.08.002 16938456

[6] Trifaró JM, Gasman S, Gutiérrez LM. Cytoskeletal control of vesicle transport and exocytosis in chromaffin cells. Acta Physiologica. 2008;192(2):165–172. 18021329 10.1111/j.1748-1716.2007.01808.x

[7] Brown SS. Cooperation Between Microtubule- and Actin-Based Motor Proteins. Annual Review of Cell and Developmental Biology. 1999;15(1):63–80. doi: 10.1146/annurev.cellbio.15.1.63 10611957

[8] Rosé SD, Lejen T, Casaletti L, Larson RE, Pene TD, Trifaró JM. Myosins II and V in chromaffin cells: myosin V is a chromaffin vesicle molecular motor involved in secretion. Journal of Neurochemistry. 2003;85(2):287–298. doi: 10.1046/j.1471-4159.2003.01649.x 12675905

[9] Rudolf R, Kögel T, Kuznetsov SA, Salm T, Schlicker O, Hellwig A, et al. Myosin Va facilitates the distribution of secretory granules in the F-actin rich cortex of PC12 cells. Journal of Cell Science. 2003;116(7):1339–1348. doi: 10.1242/jcs.00317 12615975

[10] Ñeco P, Giner D, Del Mar Francés M, Viniegra S, Gutiérrez LM. Differential participation of actin- and tubulin-based vesicle transport systems during secretion in bovine chromaffin cells. European Journal of Neuroscience. 2003;18(4):733–742. doi: 10.1046/j.1460-9568.2003.02801.x 12924999

[11] Papadopulos A, Gomez GA, Martin S, Jackson J, Gormal RS, Keating DJ, et al. Activity-driven relaxation of the cortical actomyosin II network synchronizes Munc18-1-dependent neurosecretory vesicle docking. Nature Communications. 2015;6. doi: 10.1038/ncomms7297 25708831

[12] Tomatis VM, Papadopulos A, Malintan NT, Martin S, Wallis T, Gormal RS, et al. Myosin VI small insert isoform maintains exocytosis by tethering secretory granules to the cortical actin. Journal of Cell Biology. 2013;200(3):301–320. doi: 10.1083/jcb.201204092 PMC3563687 23382463

[13] Trifaró JM, Bader MF, Doucet JP. Chromaffin cell cytoskeleton: its possible role in secretion. Canadian Journal of Biochemistry and Cell Biology. 1985;63(6):661–679. doi: 10.1139/o85-084 2994861

[14] Gutiérrez LM, Villanueva J. The role of F-actin in the transport and secretion of chromaffin granules: an historic perspective. Pflügers Archiv—European Journal of Physiology. 2018;470(1):181–186. doi: 10.1007/s00424-017-2040-9 28730385 PMC5748413

[15] Oheim M, Stühmer W. Tracking chromaffin granules on their way through the actin cortex. European Biophysics Journal. 2000;29(2):67–89. doi: 10.1007/s002490050253 10877017

[16] Giner D, López I, Villanueva J, Torres V, Viniegra S, Gutiérrez LM. Vesicle movements are governed by the size and dynamics of F-actin cytoskeletal structures in bovine chromaffin cells. Neuroscience. 2007;146(2):659—669. doi: 10.1016/j.neuroscience.2007.02.039 17395387

[17] Meunier FA, Gutiérrez LM. Captivating New Roles of F-Actin Cortex in Exocytosis and Bulk Endocytosis in Neurosecretory Cells. Trends in Neurosciences. 2016;39(9):605–613. doi: 10.1016/j.tins.2016.07.003 27474993

[18] Porat-Shliom N, Porat-Shliom N, Milberg O, Milberg O, Masedunskas A, Masedunskas A, et al. Multiple roles for the actin cytoskeleton during regulated exocytosis. Cellular and molecular life sciences: CMLS. 2013;70(12):2099–2121. doi: 10.1007/s00018-012-1156-5 22986507 PMC4052552

[19] Maucort G, Kasula R, Papadopulos A, Nieminen TA, Rubinsztein-Dunlop H, Meunier FA. Mapping Organelle Motion Reveals a Vesicular Conveyor Belt Spatially Replenishing Secretory Vesicles in Stimulated Chromaffin Cells. PLOS ONE. 2014;9(1):1–9. doi: 10.1371/journal.pone.0087242 24489879 PMC3906151

[20] Gurel P, Hatch A, Higgs H. Connecting the Cytoskeleton to the Endoplasmic Reticulum and Golgi. Current Biology. 2014;24(14):R660–R672. doi: 10.1016/j.cub.2014.05.033 25050967 PMC4174561

[21] Smith DA, Simmons RM. Models of Motor-Assisted Transport of Intracellular Particles. Biophysical Journal. 2001;80(1):45—68. doi: 10.1016/S0006-3495(01)75994-2 11159382 PMC1301213

[22] Bressloff PC. Stochastic processes in cell biology. vol. 41. Springer; 2014.

[23] Hadeler KP. Topics in Mathematical Biology by Karl Peter Hadeler. Lecture Notes on Mathematical Modelling in the Life Sciences. Cham: Springer International Publishing: Imprint: Springer; 2017.

[24] White D, de Vries G, Dawes A. Microtubule Patterning in the Presence of Stationary Motor Distributions. Bulletin of mathematical biology. 2014;76(8):1917–1940. doi: 10.1007/s11538-014-9991-1 25033782

[25] Chen W, Zhou W, Xia T, Gu X. A Computational Analysis Framework for Molecular Cell Dynamics: Case-Study of Exocytosis. PLOS ONE. 2012;7(7):1–10. doi: 10.1371/journal.pone.0038699 22808014 PMC3394804

[26] Mayorga LS, Verma M, Hontecillas R, Hoops S, Bassaganya-Riera J. Agents and networks to model the dynamic interactions of intracellular transport. Cellular Logistics. 2017;7(4):e1392401. doi: 10.1080/21592799.2017.1392401 29296512 PMC5739099

[27] Klann M, Koeppl H, Reuss M. Spatial Modeling of Vesicle Transport and the Cytoskeleton: The Challenge of Hitting the Right Road. PLOS ONE. 2012;7(1):1–15. doi: 10.1371/journal.pone.0029645 22253752 PMC3257240

[28] Birbaumer M, Schweitzer F. Agent-based modeling of intracellular transport. The European Physical Journal B. 2011;82(245).

[29] Jarukanont D, Bonifas Arredondo I, Femat R, Garcia ME. Vesicle Motion during Sustained Exocytosis in Chromaffin Cells: Numerical Model Based on Amperometric Measurements. PloS one. 2015;10(12):e0144045–e0144045. doi: 10.1371/journal.pone.0144045 26675312 PMC4699451

[30] Oelz DB. Quasi-steady-state reduction of a model for cytoplasmic transport of secretory vesicles in stimulated chromaffin cells. Journal of Mathematical Biology. 2021;82(4):29. doi: 10.1007/s00285-021-01583-5 33661393

[31] Bressloff PC. Stochastic Processes in Cell Biology by Paul C. Bressloff. 1st ed. Interdisciplinary Applied Mathematics, 41. Cham: Springer International Publishing: Imprint: Springer; 2014.

[32] Papadopulos A. Membrane shaping by actin and myosin during regulated exocytosis. Molecular and Cellular Neuroscience. 2017;84:93–99. doi: 10.1016/j.mcn.2017.05.006 28536001

[33] Bezanson J, Edelman A, Karpinski S, Shah VB. Julia: A Fresh Approach to Numerical Computing. SIAM Review. 2017;59(1):65–98. doi: 10.1137/141000671

[34] Efimov A, Kharitonov A, Efimova N, Loncarek J, Miller PM, Andreyeva N, et al. Asymmetric CLASP-Dependent Nucleation of Noncentrosomal Microtubules at the trans-Golgi Network. Developmental Cell. 2007;12(6):917–930. doi: 10.1016/j.devcel.2007.04.002 17543864 PMC2705290

[35] Rivero S, Cardenas J, Bornens M, Rios RM. Microtubule nucleation at the cis-side of the Golgi apparatus requires AKAP450 and GM130. The EMBO Journal. 2009;28(8):1016–1028. doi: 10.1038/emboj.2009.47 19242490 PMC2683699

[36] Wang T, Martin S, Papadopulos A, Harper CB, Mavlyutov TA, Niranjan D, et al. Control of Autophagosome Axonal Retrograde Flux by Presynaptic Activity Unveiled Using Botulinum Neurotoxin Type A. Journal of Neuroscience. 2015;35(15):6179–6194. doi: 10.1523/JNEUROSCI.3757-14.2015 25878289 PMC4787026

[37] Joensuu M, Padmanabhan P, Durisic N, Bademosi ATD, Cooper-Williams E, Morrow IC, et al. Subdiffractional tracking of internalized molecules reveals heterogeneous motion states of synaptic vesicles. Journal of Cell Biology. 2016;215(2):277–292. doi: 10.1083/jcb.201604001 27810917 PMC5080683

[38] Crivellato E, Nico B, Ribatti D. The Chromaffin Vesicle: Advances in Understanding the Composition of a Versatile, Multifunctional Secretory Organelle. The Anatomical Record. 2008;291(12):1587–1602. doi: 10.1002/ar.20763 19037853

[39] Alon U. An introduction to systems biology: design principles of biological circuits. CRC press; 2019.

