# Supplementary material for: Spatial redistribution of neurosecretory vesicles upon stimulation accelerates their directed transport to the plasma membrane
Source: PLoS One. 2022 Mar 16;17(3):e0264521. doi: 10.1371/journal.pone.0264521 (PMC8926195; doi:10.1371/journal.pone.0264521)
Supplement: S1 File — (PDF) [file pone.0264521.s001.pdf]

S1 Steady state solution of the minimal 4-state model.

The steady state solution of the system (1) with $\zeta > 0$ satisfies the following equations,

$$G = \frac{\mu \bar{G}}{\kappa_{gr} + \mu} + \frac{\kappa_{lg}}{\kappa_{gr} + \mu} L \; , \tag{8}$$

$$R = \frac{\kappa_{gr}}{\kappa_{rc}} G \; , \tag{9}$$

$$L = \frac{\kappa_{cl}}{\kappa_{lg}} C \; , \tag{10}$$

$$C = \frac{\kappa_{rc}}{\kappa_{cl} + \zeta} R \; . \tag{11}$$

We can now express C and L in terms of G as follows,

$$C = \frac{\kappa_{gr}}{\kappa_{cl} + \zeta} G \; , \tag{12}$$

$$L = \frac{\kappa_{gr} \kappa_{cl}}{\kappa_{lg}(\kappa_{cl} + \zeta)} G \; . \tag{13}$$

Now we can combine (8), (13) to get the steady state solution for G in terms of $\bar{G}$,

$$G = \bar{G} \frac{1}{1 + \frac{\kappa_{gr}}{\mu} \cdot \frac{1}{1 + \frac{\kappa_{cl}}{\zeta}}} \; .$$

This is the steady state for the population size in state G. As mentioned in section Minimal 4-state model it converges to $\bar{G}$ in the limit when the rate μ is large. This complements the steady state abundances (9),(12) and (13).

S2 Governing Equations.

We provide below the full system of coupled differential equations describing the Markov state model visualised in Fig 5A and Fig 5B above.

$$\begin{aligned}
\dot{R}_1(t) &= \kappa_{fr}^1 F_i + \kappa_{cr}^1 C_1 - \left(\tau + \kappa_{rf}^1 + \kappa_{dc}^1\right) R_1 \\
\dot{R}_2(t) &= \kappa_{fr}^2 F_2 + \kappa_{cr}^2 C_2 + \tau R_1 - \left(\tau + \kappa_{rf}^2 + \kappa_{dc}^2\right) R_2 \\
\dot{R}_3(t) &= \kappa_{fr}^3 F_3 + \kappa_{cr}^3 C_3 + \tau R_2 - \left(\kappa_{rf}^3 + \kappa_{dc}^3\right) R_3 \\
\dot{C}_1(t) &= \kappa_{fc}^1 F_1 + \kappa_{dc}^1 (R_1 + L_1) + \mu(\bar{C} - C_1)_+ - \left(\kappa_{cf}^1 + \kappa_{cd}^1\right) C_1 \\
\dot{C}_2(t) &= \kappa_{fc}^2 F_2 + \kappa_{dc}^2 (R_2 + L_2) - \left(\kappa_{cf}^2 + \kappa_{cd}^2\right) C_2 \\
\dot{C}_3(t) &= \kappa_{fc}^3 F_3 + \kappa_{dc}^3 (R_3 + L_3) - \left(\kappa_{cf}^3 + \kappa_{cd}^3 + \zeta\right) C_3 \\
\dot{F}_1(t) &= \kappa_{df}^1 \left(R_1 + L_1\right) + \kappa_{cf}^1 C_i - \left(\kappa_{fc}^1 + \kappa_{fd}^1\right) F_1 + \delta(F_2 - F_1) \\
\dot{F}_2(t) &= \kappa_{df}^2 \left(R_2 + L_2\right) + \kappa_{cf}^2 C_2 - \left(\kappa_{fc}^2 + \kappa_{fd}^2\right) F_2 + \delta(F_3 - F_2) + \delta(F_1 - F_2) \\
\dot{F}_3(t) &= \kappa_{df}^3 \left(R_3 + L_3\right) + \kappa_{cf}^3 C_3 - \left(\kappa_{fc}^3 + \kappa_{fd}^3\right) F_3 + \delta(F_2 - F_3) \\
\dot{L}_1(t) &= \kappa_{fl}^1 F_1 + \kappa_{cl}^1 C_1 + \tau L_2 - \left(\kappa_{lf}^1 + \kappa_{dc}^1\right) L_1 \\
\dot{L}_2(t) &= \kappa_{fl}^2 F_2 + \kappa_{cl}^2 C_2 + \tau L_3 - \left(\tau + \kappa_{lf}^2 + \kappa_{dc}^2\right) L_2 \\
\dot{L}_3(t) &= \kappa_{fl}^3 F_3 + \kappa_{cl}^3 C_3 - \left(\tau + \kappa_{lf}^3 + \kappa_{dc}^3\right) L_3
\end{aligned}$$

S3 Steady state distributions, absolute values and uncertainty

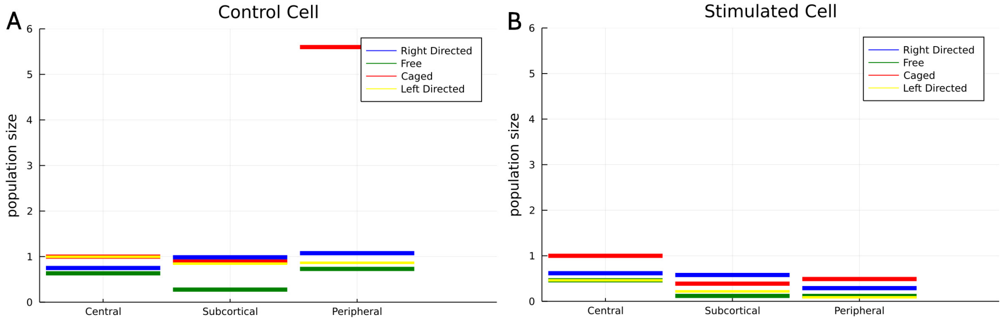

Fig S1. Simulated steady state probability distribution of vesicles with transition rates (5) and (6) in control (A) and upon stimulation (B), visualised with absolute values when normalised such that $C1 = 1$.

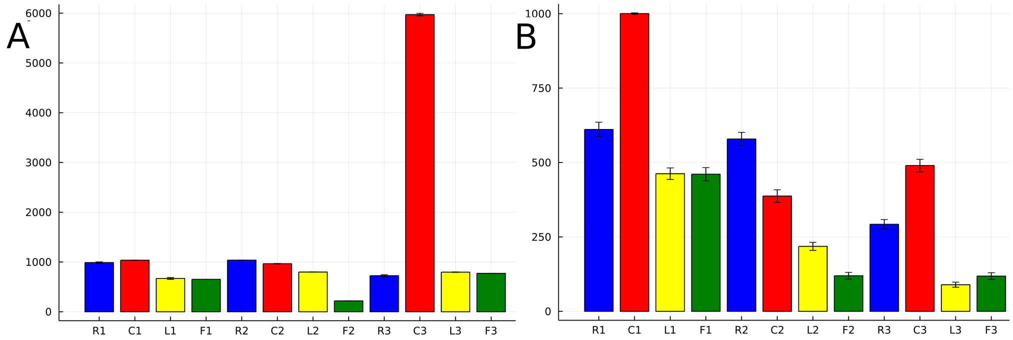

Fig S2. Stochastic simulation of vesicle distributions at steady state in control (A) and upon stimulation (B). Dumbbell-shaped bars show standard deviation. For the stochastic simulation we up-scaled all parameters of dimension #vesicles by the factor 1000 in order to make the total vesicle counts comparable to observed vesicle counts for single chromaffin cells [1].

S4 Modification of the model introducing crowding

In [2] it was also reported that transition rates from the pool of free vesicles into directed transport accelerate three-fold upon stimulation (Fig 2A). This effect can't be predicted by our model which relies on the assumption of constant transition rates. In this supplementary section we present a modification of our model visualised in Fig 5A and Fig 5B suggesting that specifically the central cytoskeleton network in control cells is limited by its carrying capacity. We show that this hypothetical mechanism is consistent with the observations (Fig 2A).

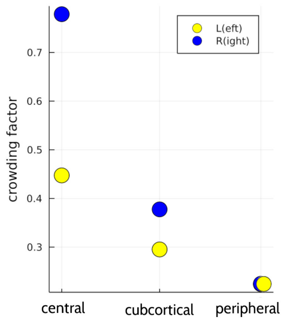

Fig S3. Crowding correction factors (14) in control.

We hypothesise that the observed increase in the average free-to-directed transition rate upon stimulation is a consequence of the global decrease in vesicle abundance occurring at stimulation. We therefore suggest that in control transitions are slowed down by crowding of vesicles undergoing directed transport.

Description	Symbol	Value	Reference
Repression (crowding) coefficients	$\hat{R}_i$, $\hat{L}_i$ $(i = 1, 2, 3)$	0.2	Estimated

Table S1. Parameter values

We use Hill functions (for a repressor [3]) to model the limiting effect of carrying capacities on all other transition rates into directed transport by the following correction factors,

$$\alpha_i(R_i) = \frac{1}{1 + \frac{(R_i - R_i^{\zeta_{\text{stim}}})_+}{\hat{R}_i}} \quad \text{and} \quad \beta_i(L_i) = \frac{1}{1 + \frac{(L_i - L_i^{\zeta_{\text{stim}}})_+}{\hat{L}_i}} \quad \text{for} \quad i = 1, 2, 3 \,. \tag{14}$$

The Hill functions are parametrised by the repression coefficients $\hat{R}_i$ and $\hat{L}_i$ $(i = 1, 2, 3)$. To ensure that this modification does not alter the steady state population sizes in stimulation, $R_i^{\zeta_{\text{stim}}}$ and $L_i^{\zeta_{\text{stim}}}$, we include these values in the correction factors (14) taking only the positive part of $R_i - R_i^{\zeta_{\text{stim}}}$ and $L_i - L_i^{\zeta_{\text{stim}}}$, respectively.

It obviously appears problematic to include steady state abundances in stimulation in the correction factors. To remove these dependencies one might rewrite the correction factors as a classical Hill function with two constants, namely $\alpha_i(R_i) = \mu_i/(1 + R_i/\nu_i)$ (for $R_i > R_i^{\zeta_{\text{stim}}}$) and analogous expressions for β_i. Nevertheless, such rewriting of the correction is not required to run the simulations and it would render our choice of parameters less transparent. We therefore keep the notation used in (14).

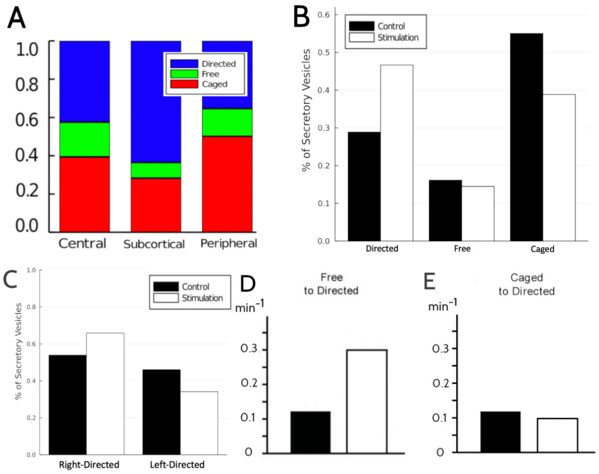

Fig S4. Steady state simulation results for model with carrying capacities: **A** Intra-compartmental proportions in stimulated cells. **B** Percentages of secretory vesicles in each motile state. **C** Percentages of secretory vesicles undergoing direction motion (right vs left). **D** Aggregated transition rates from free to directed motion. **E** Aggregated transition rates from caged to directed motion.

Note that applying the correction factors (14) to the transition rates (5) would also slow down transitions from the pool of caged vesicles into transport states. Reported transition rates from caged into directed motion (averaged among spatial compartments), however, only exhibit minor variation between control and stimulation (Fig 2B). We speculate that for caged vesicles the speed-up in response to less crowding along transport fibres upon stimulation might be compensated by faster turnover of the actomyosin cortex upon stimulation [4] which could prevent a large fraction of caged vesicles from binding to fibre-motor-protein complexes.

As a consequence we keep the peripheral caged-to-directed transition rates unmodified, while rescaling all rates listed in (5) as follows

$$\begin{array}{llll} \bar{\kappa}^1_{fr} = \kappa^1_{fr}\,\alpha_1\ , & \bar{\kappa}^1_{fl} = \kappa^1_{fl}\,\beta_1\ , & \bar{\kappa}^1_{cr} = \kappa^1_{cr}\,\alpha_1\ , & \bar{\kappa}^1_{cl} = \kappa^1_{cl}\,\beta_1\ , \\ \bar{\kappa}^2_{fr} = \kappa^2_{fr}\,\alpha_2\ , & \bar{\kappa}^2_{fl} = \kappa^2_{fl}\,\beta_2\ , & \bar{\kappa}^2_{cr} = \kappa^2_{cr}\,\alpha_2\ , & \bar{\kappa}^2_{cl} = \kappa^2_{cl}\,\beta_2\ , \\ \bar{\kappa}^3_{fr} = \kappa^3_{fr}\,\alpha_3\ , & \bar{\kappa}^3_{fl} = \kappa^3_{fl}\,\beta_3\ , & \bar{\kappa}^3_{cr} = \kappa^3_{cr}\ , & \bar{\kappa}^3_{cl} = \kappa^3_{cl}\ . \end{array} \qquad (15)$$

In the absence of more detailed data, and since we only seek to provide a proof of concept for the idea that crowding can explain the 3-fold speed-up of transitions from free to directed (Fig 2A) we choose a single constant for the repression coefficients (see Table S1).

We fit a single, spatially uniform, repressor coefficient (Table 1) in a way such that - at steady state - the average free-to-directed transition rate is $0.126\,\mathrm{min}^{-1}$ in control ($0.29\,\mathrm{min}^{-1}$ in stimulation) as compared to $0.27\,\mathrm{min}^{-1}$ (control) ($0.3\,\mathrm{min}^{-1}$ in stimulation) for the model with constant transition rates. The model with non-linear transition rates modelling crowding therefore allows to reproduce the 3-fold speed-up of transitions from free into directed motion (Fig S4D) observed experimentally (Fig 2A).

Note that we do not reassess the directional parameters ρ_i, $i = 1, 2, 3$ with the modified transition rates. With these parameters the modified model predicts the ratio of outward vs inward transport (Fig S4C) as well as the increase of total transport (Fig S4B) almost equally well as the original model.

The correction factors with the uniform repressor coefficients listed in Table 1 are close to one in the central compartment, indicating that crowding in control is stronger in the periphery of the cell (the exact correction factors are visualised in supplementary

Fig S3). This reflects that most variation of vesicle abundance between control and stimulation is in the periphery of the cell, since the steady state distribution of vesicles in control is predominantly characterised by an accumulation of vesicles close to the cortex (see steady state distributions in absolute numbers shown in supplementary Fig S1).

We reiterate that the modification of this model through carrying capacities of the transport network is entirely speculative. We report it in the supplementary material setion of this study to illustrate that even the observed increase in the transition rate free → directed (Fig 2A) in stimulated cells, which cannot be explained by spatial redistribution alone, in principle can be explained as a purely mechanistic effect and doesn't require a specific biochemical feedback. Nevertheless, more experimental data would be required to make a definite statement.

References

1. Crivellato E, Nico B, Ribatti D. The Chromaffin Vesicle: Advances in Understanding the Composition of a Versatile, Multifunctional Secretory Organelle. The Anatomical Record. 2008;291(12):1587–1602. doi:https://doi.org/10.1002/ar.20763.

2. Maucort G, Kasula R, Papadopulos A, Nieminen TA, Rubinsztein-Dunlop H, Meunier FA. Mapping Organelle Motion Reveals a Vesicular Conveyor Belt Spatially Replenishing Secretory Vesicles in Stimulated Chromaffin Cells. PLOS ONE. 2014;9(1):1–9. doi:10.1371/journal.pone.0087242.

3. Alon U. An introduction to systems biology: design principles of biological circuits. CRC press; 2019.

4. Papadopulos A. Membrane shaping by actin and myosin during regulated exocytosis. Molecular and Cellular Neuroscience. 2017;84:93–99. doi:https://doi.org/10.1016/j.mcn.2017.05.006.
